# Impact of Deposition Power and Gas Flow Ratio on the Tribological Properties of Titanium Vanadium Nitride Thin Films

**DOI:** 10.3390/mi14091788

**Published:** 2023-09-19

**Authors:** Kamlesh V. Chauhan, Sushant Rawal, Nicky P. Patel, Dattatraya G. Subhedar

**Affiliations:** 1 CHAMOS Matrusanstha Department of Mechanical Engineering, Chandubhai S. Patel Institute of Technology (CSPIT), Charotar University of Science and Technology (CHARUSAT), Changa 388421, Gujarat, India; 2 McMaster Manufacturing Research Institute (MMRI), Department of Mechanical Engineering, McMaster University, 1280 Main Street West, Hamilton, ON L8S4L7, Canada

**Keywords:** titanium vanadium nitride, sputtering, tribology, friction, wear

## Abstract

Magnetron sputtering was used for producing titanium vanadium nitride (TiVN) coatings on brass substrates. In this research, we investigate how changing the sputtering power and nitrogen:argon (N_2_:Ar) gas ratio affects the structural and tribological properties of TiVN coatings. A scanning electron microscope (SEM) was used to examine TiVN coating surface morphology. Both variants showed a gradual increase in the intensity of the TiVN coatings’ (111) and (222) peaks. The TiVN coatings’ tribological properties were examined using a pin-on-disc tribometer with varying loads, speeds, and sliding distances. The wear rates of TiVN-coated brass pins were in the range of 2.5 × 10^−4^ to 9.14 × 10^−4^ mm^3^/Nm depending on load, sliding distance, and gas ratio variation, when compared to the wear rates of TiVN-coated brass pins deposited at various powers, which ranged from 1.76 × 10^−3^ to 5.87 × 10^−3^ mm^3^/Nm.

## 1. Introduction

Many facets of our lives rely on coating technologies. Numerous coatings and thin films have been designed for use on a wide variety of products, including those used in the consumer clothing goods and pharmaceutical industries, as well as on machinery used in manufacturing, vehicles, and architectural elements. Bulk materials and various surfaces can be protected, improved, or endowed with new functions and qualities by having a thin film or coating deposited on top of them as an external film [1]. In present industrial systems, a significant focus is on the development and manufacturing of materials that possess a diminished coefficient of friction and reduced wear characteristics, suitable for diverse operational contexts. The field of coating technology has seen a rapid growth in response to the significant industrial need for friction control and wear resistance. This demand is further fueled by the development of supporting technologies that facilitate the creation of novel coatings with acceptable tribological performance and mechanical attributes [2].

Titanium-based metal nitride coatings, namely, those comprised of TiN, are widely utilized in several industries, including automobile manufacturing, aerospace engineering, medical implantation, and tool fabrication. The extensive use of these coatings may be largely ascribed to their excellent properties, including amazing wear resistance, friction reduction capabilities, corrosion resistance, and significant hardness. Various techniques are employed for the application of these coatings, including magnetron sputtering, chemical vapor deposition, and other similar methodologies [3,4,5,6,7,8]. In recent years, there has been much study and industry focus on the advancement of complex ternary nitride coatings. This attention stems from the need to enhance film characteristics such as enhanced hardness, superior wear resistance, exceptional corrosion protection, and elevated melting point, among others. The addition of a third element, such as vanadium (V), molybdenum (Mo), aluminum (Al), etc., to titanium nitride (TiN) in order to generate ternary alloys has been seen to improve the wear resistance and reduce the friction of the resultant films [9,10,11]. The addition of a limited amount of the third substance induces alterations in the morphology, structure, and bonding characteristics of the film coating [12,13]. The ternary alloys TixMoyN and TixVyN possess the capability to reduce the coefficient of friction under dry sliding circumstances by the production of lubricous oxides of Mo and V, respectively. TixVyN exhibits enhanced hardness and strength as compared to its constituent binary nitride constituents, hence facilitating the reduction in wear [14].

There exists a lack of literature pertaining to the examination of several characteristics of titanium vanadium nitride (TiVN) coatings. The objective of this study was to employ the reactive magnetron sputtering technique in order to develop titanium vanadium nitride (TiVN) coatings on brass substrates. This study effort investigates the impact of the nitrogen:argon (N_2_:Ar) gas ratio and sputtering power on the structural and tribological characteristics of TiVN coatings.

## 2. Materials and Methods

The deposition of titanium vanadium nitride (TiVN) coatings was carried out using DC magnetron sputtering within a cylindrical chamber that was specifically developed by Excel Instruments, a company based in India. Two different targets with a diameter of 50.8 mm each, composed of titanium and vanadium with a purity level of 99.99%, were employed in the sputtering procedure. The chamber was originally subjected to an evacuation process, resulting in a pressure of about 5 × 10^−4^ Pa. This evacuation was achieved by utilizing a turbo molecular pump, which was supported by a rotary pump. Subsequently, a mixture of high-quality argon and nitrogen gases, with a purity level of 99.99%, was introduced into the chamber. The regulation and maintenance of gas flow were achieved by utilizing a mass flow controller (MFC) manufactured by ALICAT instruments, Tucson, AZ, USA. The sputtering pressure was adjusted to 1.5 Pa, while maintaining a constant power of 275 W for the titanium and vanadium targets in the initial batch of samples. The substrates were maintained at a constant temperature of 500 °C during the deposition process. The duration of each deposition was 60 min, and the distance between the target and substrate was set at 50 mm. The gas ratio of nitrogen to argon (N_2_:Ar), as measured in standard cubic centimeters per minute (sccm), exhibited variations among different samples. Specifically, the recorded ratios were 08:32, 08:12, 10:10, 08:02, and 08:00 sccm for the samples named TiVN-32, TiVN-12, TiVN-10, TiVN-02, and TiVN-00, respectively. In the subsequent set of samples, the gas ratio of nitrogen to argon (N_2_:Ar) was maintained at 08:32 standard cubic centimeters per minute (sccm). The power applied to the titanium (Ti) target was set at 275 W, while the power applied to the vanadium (V) target was systematically adjusted to 325, 350, 400, and 450 W, resulting in the corresponding sample names of TiVN-325, TiVN-350, TiVN-400, and TiVN-450, respectively.

The crystal structure of TiVN coatings was investigated using X-ray diffractometry (XRD) with a Bruker Model D2 Phaser instrument. The microstructure of the coatings was examined with a scanning electron microscope (SEM) using the model EVO-18 manufactured by ZEISS. A pin-on-disc tribometer (Ducom) was utilized to conduct wear and coefficient-of-friction studies under ambient conditions, without the use of lubrication. The experimental setup for the tribological system consisted of a pin-on-disc configuration. The pin used in the testing was made of brass (grade 1, IS-319) and had a diameter of 12 mm and a height of 30 mm. It was in contact with a disc that had a diameter of 165 mm and a height of 8 mm. Tribological experiments were performed to evaluate the performance of both uncoated and TiVN-coated brass pins when subjected to rotational motion against a disc composed of En-31 steel (BS-970-1955), which had been hardened to a hardness level of 60 HRc. These tests were carried out under ambient room temperature conditions. During the experimental trials, the discs were subjected to rotational speeds of 500 revolutions per minute (rpm), while the sliding distance was in a range of 628 to 785 m. The applied loads were 10, 20, 30, and 40 N, respectively.

## 3. Results and Discussion

**Figure 1:**
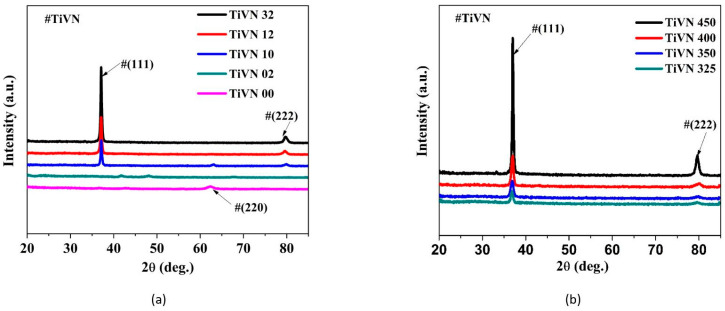
XRD patterns of titanium vanadium nitride (TiVN) coatings deposited at different (**a**) nitrogen:argon (N_2_:Ar) gas ratios and (**b**) vanadium power levels.

The texture development of TiVN coatings was assessed using an X-ray diffractometer (Bruker, Bremen, Germany, Model D2 Phaser) Bragg Brentano geometry with Cu-K_α_ radiation of wavelength 1.54 Å. Figure 1a displays the X-ray diffraction (XRD) patterns of TiVN coatings that were deposited using varying gas ratios. A slightly crystalline (220) peak of TiVN was detected in sample TiVN-00 under a pure nitrogen atmosphere with a nitrogen:argon (N_2_:Ar) gas ratio of 08:00. The observation of the development of (111) and (222) peaks of TiVN was limited to the rise in argon flow rate from 0 to 10 sccm, specifically at a N_2_:Ar gas ratio of 10:10 for sample TiVN-10. The present study reveals that an increase in the argon gas flow rate leads to a corresponding rise in the strength of the (111) and (222) peaks. The potential explanation for this phenomenon is in the positive correlation between the quantity of argon gas and the likelihood of a reaction occurring between argon, titanium, and vanadium. Consequently, this enhanced possibility leads to a greater production of the TiVN phase. Moreover, an elevated concentration of argon leads to an augmentation in the frequency of collisions between argon ions and titanium and vanadium ions. Consequently, this facilitates the formation of a crystalline phase in the coating. Figure 1b illustrates the X-ray diffraction (XRD) patterns of titanium vanadium nitride (TiVN) coatings that were coated using varying vanadium powers. The presence of (111) and (222) orientations in all samples indicates the existence of TiVN coating. The observed intensities of the (111) and (222) peaks exhibit a positive correlation with the power levels applied to the vanadium samples, namely, from TiVN-325 to TiVN-450. The quantity of ejected atoms that reach the surface surged with increasing deposition power, which in turn affected the high-energy particle bombardment of the thin layer that was growing. These elements supply thermal energy to the atoms that are deposited, thus facilitating their mobility on the substrate and ultimately enhancing the formed coatings’ crystal structure. In their work, Deeleard et al. conducted studies involving the manipulation of the sputtering current applied to the vanadium target. They observed a distinct preference for the (111) crystallographic orientation, which was found to be positioned between the (111) textures of TiN (JCPDS 87-0633) and VN (JCPDS 89-5265). The intensity of this preferred orientation was found to increase as the sputtering current was raised, suggesting a corresponding enhancement in both the crystallinity and thickness of the films [15]. This research achieved success in the synthesis of a singular compound, specifically TiVN coatings (JCPDS 89-5212), which exhibited distinct (220), (111), and (222) textures. This accomplishment was attained by varying the nitrogen:argon (N_2_:Ar) gas ratios and power levels. It is noteworthy that previous studies predominantly documented the formation of separate TiN and VN compounds.

The crystallite size of the films was determined from Scherrer’s formula [16]

(1) $$D=\frac{0.94\lambda }{\Delta \omega cos\theta_{B}}$$

where *θ_B_* denotes the Bragg angle, Δ*ω* signifies the full width at half maximum (FWHM) of the peak, *λ* indicates the wavelength, and *D* denotes the average crystallite size.

**Figure 2:**
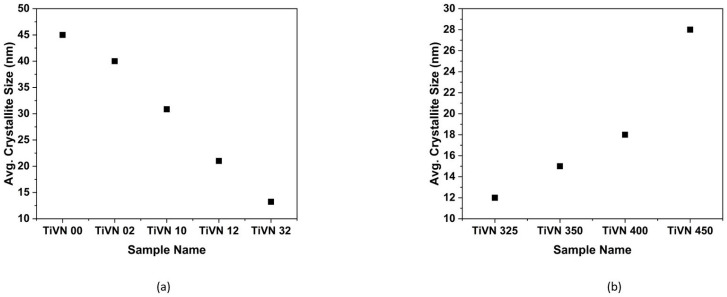
Average crystallite size of titanium vanadium nitride (TiVN) coatings deposited at different (**a**) nitrogen:argon (N_2_:Ar) gas ratios and (**b**) vanadium power levels.

The study revealed that an increase in the nitrogen:argon (N_2_:Ar) gas ratio resulted in a decrease in the average crystallite size of TiVN coatings. The aforementioned XRD data are consistent with these findings. The study revealed a drop in the average crystallite size of TiVN coatings, from about 45 nm for sample TiVN-00 to 13.23 nm for sample TiVN-32, while the nitrogen:argon (N_2_:Ar) gas ratio was adjusted from 08:00 to 08:32, respectively. Figure 2a,b show the average crystallite size for thin films deposited at varying gas flow rate and sputtering power levels. According to the findings of R. Mishra et al., it was observed that the hardness exhibited a general tendency to rise as the grain size decreased [17]. In their study, Yeung et al. conducted a deposition process to create TiVN coatings at varying nitrogen pressures of 0.053 and 0.128 Pa. The researchers found a notable rise in the grain size of the TiVN coatings, which transitioned from an initial size of 200 nm to a final size of 350 nm [18]. In our research, we deliberately maintained a lower nitrogen flow rate while increasing the argon flow rate. This deliberate adjustment may have led to a slight increase in the average crystallite size of the TiVN coatings. The observed average grain size exhibited a notable increase from 12 to 28 nm as a consequence of enhancing the vanadium sputtering power in the case of the TiVN-325 and TiVN-450 samples. The literature has documented a comparable rise in the average crystallite size, from 20 to 24 nm, as a result of an increase in vanadium target power [15].

**Figure 3:**
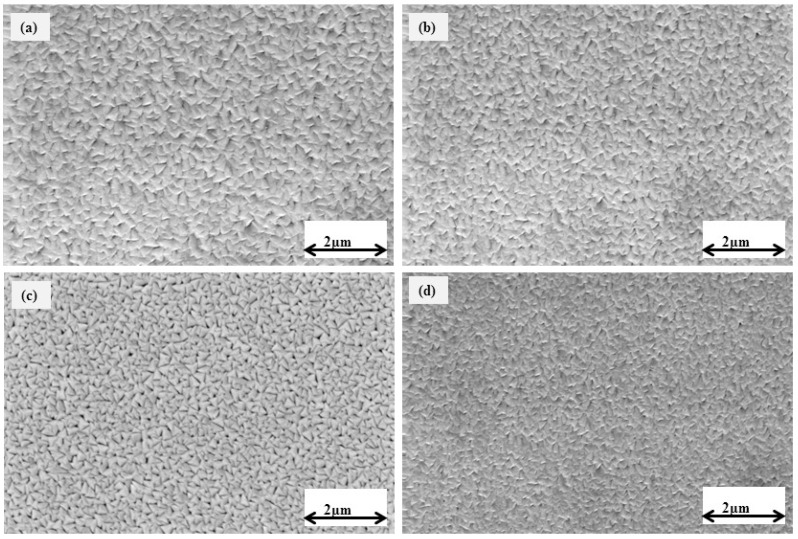
SEM images of titanium vanadium nitride (TiVN) coatings deposited at different nitrogen:argon (N_2_:Ar) gas ratios: (**a**) 08:02, (**b**) 08:12, (**c**) 10:10, and (**d**) 08:32.

**Figure 4:**
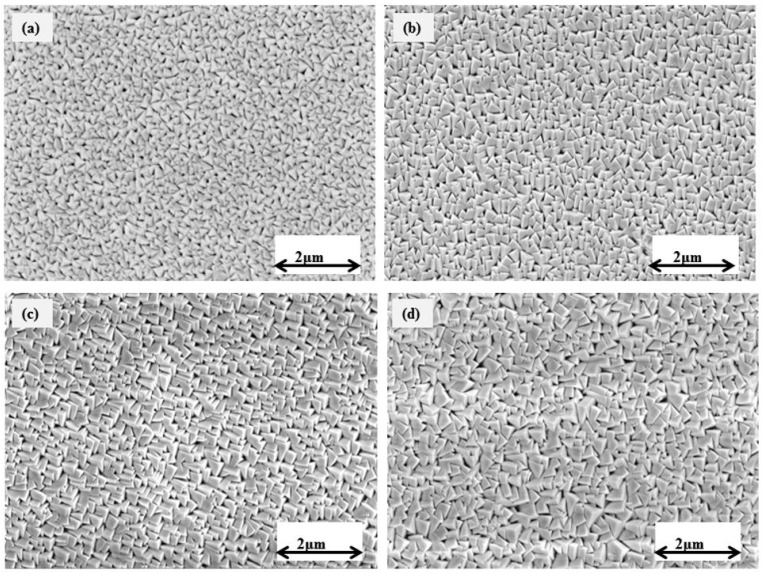
SEM morphology of the titanium vanadium nitride (TiVN) films deposited at different V sputtering power: (**a**) 325, (**b**) 350, (**c**) 400, and (**d**) 450 W.

Figure 3 displays the scanning electron microscopy (SEM) images of TiVN coatings that were formed using different ratios of nitrogen to argon (N_2_:Ar) gas. The TiVN coatings have a consistent, even, and defect-free morphology, displaying a triangular shape as evidenced by scanning electron microscopy (SEM) images. The microstructure and grain size of coatings made with a lower gas flow were found to be denser and coarser. On the other hand, the size of the grains decreased proportionally as the Ar flow rate went up. This account regarding the different growth rates and how they are linked to the species’ mean free path to the developing surface is also supported by the shape of the surface and the structural analysis. On the other hand, Figure 4 exhibits the SEM images of TiVN coatings that were deposited at different power levels of vanadium target. When the power of the coatings changed, the surface patterns of the thin films changed a lot. Also, both the number of grain boundaries and the growth of grains in the TiVN thin films increased. The XRD graphs showed that the thin films made at higher power levels had a denser structure and bigger grains.

**Figure 5:**
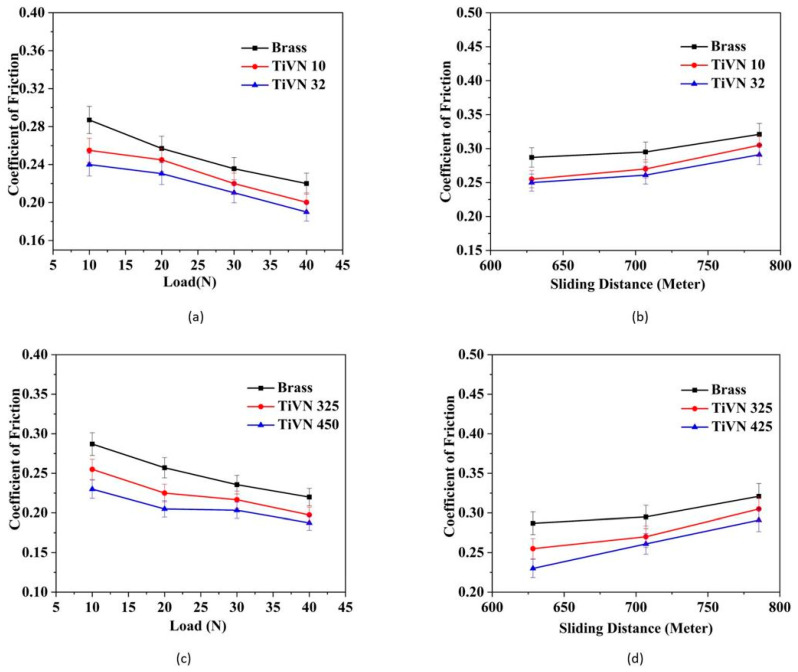
Coefficient of friction (COF) of uncoated and TiVN-coated brass pins TiVN-32 andTiVN-10 at different values of (**a**) load and (**b**) sliding distance and TiVN-325 and TiVN-450 at different values of (**c**) load and (**d**) sliding distance.

The primary objective of tribological research is to develop surface designs that effectively limit or regulate the coefficient of friction and wear. The relationship between frictional force and applied load has been empirically verified, indicating a direct proportionality. This proportionality is quantified by the coefficient of friction, denoted as indicated in Equation (2) below:

(2) $$\mu =\frac{F}{W}$$

where “*μ*” represents the coefficient of friction (COF), whereas “*F*” denotes the frictional force and “*W*” signifies the applied load [19]. Figure 5a,b display the friction coefficients of uncoated and TiVN-coated brass pins, specifically identified as TiVN-32 and TiVN-10, respectively. The coefficient of friction associated with TiVN coatings typically falls within the range of *µ* = 0.19–0.25 at room temperature, with variations influenced by factors such as load, sliding distance, and grain size. According to the data presented in Figure 5a, there is a progressive drop in the friction coefficient of the TiVN coatings as the load increases from 10 to 40 N, with values decreasing from 0.25 to 0.19. According to the data presented in Figure 5b, it can be observed that the coefficient of friction of the TiVN coatings exhibits a progressive rise from 0.255 to 0.305 as the sliding distance increases. The coefficient of friction shown by the uncoated brass pin varies between 0.22 and 0.287 under ambient temperature conditions, with the specific value dependent on factors such as load and sliding distance. It is evident that the friction coefficients of TiVN coatings are lower in comparison to those of uncoated brass. The coefficient of friction decreases as the grain size of TiVN coatings decreases. The coefficient of friction observed in the sample TiVN-10, with an average crystallite size of 28 nm, was found to be greater than that of the sample TiVN-32, which had an average crystallite size of 13 nm. According to the findings reported by Mishra et al., it has been shown that the coefficient of friction exhibits a drop as the grain size is reduced [17]. The tribology characteristics of (V, Ti)N coatings were explored by Ouyang and Sasaki. The researchers made an observation that when the load increased from 20 to 70 N, there was a decrease in the coefficient of friction of the (V, Ti)N coating from 0.81 to 0.68 [20]. A comparable pattern was seen in our study, wherein an increase in load from 10 to 40 N resulted in a drop in the friction coefficient for brass pins coated with TiVN.

Figure 5c,d display the friction coefficients of uncoated and TiVN-coated brass pins from samples TiVN-325 and TiVN-450, respectively. According to the observations made in Figure 5c, it is evident that the coefficients of friction tend to decrease as the load increases. The friction coefficients of the uncoated brass pin exhibited a drop from 0.28 to 0.22 when the applied load increased from 10 to 40 N. A minimal coefficient of friction value of 0.18 was found for the TiVN-325 sample under a load of 40 N. Figure 5d illustrates the impact of the coefficient of friction on sliding lengths with a weight of 10 N. The experimental results indicate that the coefficients of friction for both uncoated and TiVN-coated brass pins exhibit an upward trend when the sliding distance is increased. The coefficient of friction of an uncoated brass pin exhibits an increase from 0.28 to 0.32 as the sliding distance is increased. The TiVN-450 coating has the highest coefficient of friction value, measuring at 0.31, while the TiVN-325 coating demonstrates the lowest coefficient of friction value, measuring at 0.23. The tribological properties of TiVN coatings were investigated by Ouyang and Sasaki. The researchers made the observation that as the applied load is altered within the range of 20 to 100 N, there is a corresponding drop in the coefficient of friction of the coating from 0.90 to 0.57 [21]. A comparable pattern and decrease in the coefficient of friction were noted when the load increased from 10 to 40 N in the case of brass pins coated with TiVN.

The extent of wear experienced is contingent upon factors such as the hardness of the surface, the load, and the distance over which sliding occurs. The calculation for wear volume loss is determined according to Equation (3) as presented below:

(3) $$V=\frac{cLx}{H}$$

where “*c*” is a non-dimensional constant, “*L*” is the load, “*x*” is the sliding distance, and “*H*” is the hardness of the surface [19].

The wear rate is calculated as per Equation (4) given below:

(4) $$Wear\;rate=\frac{wear\times \pi \times d^{2}}{4\times Load\times SD}\;\frac{{\mathrm{mm}}^{3}}{\mathrm{Nm}}$$

where “*d*” is diameter of the pin and “*SD*” denotes the sliding distance.

**Figure 6:**
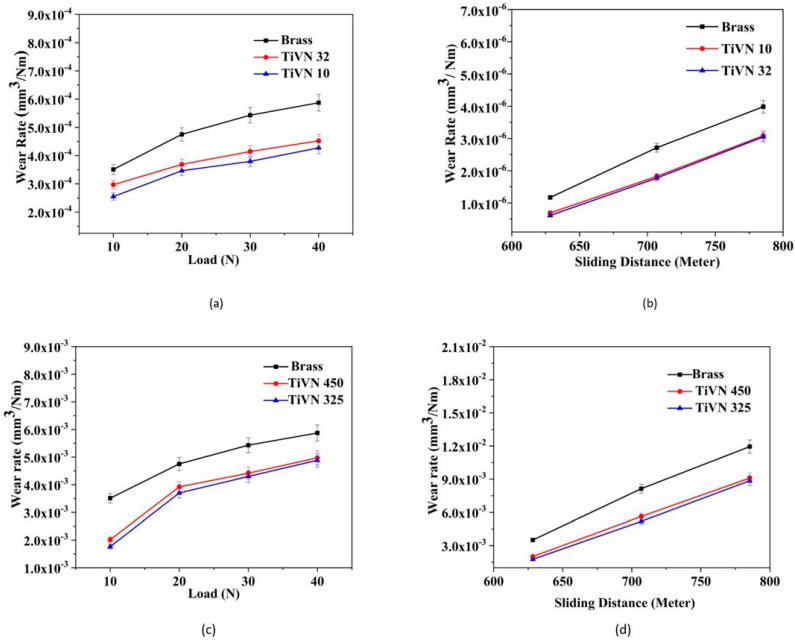
Wear rate of uncoated and TiVN-coated brass pins TiVN-32 andTiVN-10 at different values of (**a**) load and (**b**) sliding distance and TiVN-325 and TiVN-450 at different values of (**c**) load and (**d**) sliding distance.

The effects of load on the wear rate of uncoated and TiVN-coated brass pins, namely, those with sample names TiVN-32 and TiVN-10, are depicted in Figure 6a. It has been observed that the rate of wear for an uncoated brass pin is greater in comparison to a TiVN-coated brass pin. The wear rates of TiVN-32 and TiVN-10 coatings, as well as brass pins, exhibit a range of 2.54 × 10^−4^ to 5.87 × 10^−4^ mm^3^/Nm, with variations depending on the applied load. When the applied load is raised from 10 to 40 N, the rate of wear for the uncoated brass pin exhibits an increase from 3.51 × 10^−4^ to 5.87 × 10^−4^ mm^3^/Nm. Similarly, for the TiVN-10 coating, the wear rate goes up from 2.97 × 10^−4^ to 4.52 × 10^−4^ mm^3^/Nm, and for the TiVN-32 coating, it increases from 2.54 × 10^−4^ to 4.27 × 10^−4^ mm^3^/Nm.

Figure 6b compares the wear rates of uncoated and TiVN-coated brass pins (TiVN-32 and TiVN-10) as a function of sliding distance. The wear rate of an uncoated brass pin increases from 3.51 × 10^−4^ to 1.2 × 10^−3^ mm^3^/Nm as the sliding distance increases, whereas it climbs from 2.97 × 10^−4^ to 9.25 × 10^−4^ mm^3^/Nm for a TiVN-10 coating and from 2.54 × 10^−4^ to 9.14 × 10^−4^ mm^3^/Nm for a TiVN-32 coating. The literature reports that as load is raised from 20 N to 70 N, the wear rate of TiVN coatings rises from 2.06 × 10^−7^ mm^3^/Nm to 2.25 × 10^−6^ mm^3^/Nm [20]. When subjected to a load of 40 N at a rotational speed of 500 rpm, the wear rate of brass pins coated with TiVN-10 and TiVN-32 coatings exhibited a reduction of around 23% and 27%, respectively, compared to uncoated pins. In general, a reduction in particle size is associated with an increase in hardness [17]. The TiVN-32 coating has an average crystallite size of around 13 nm, whereas the TiVN-10 coating demonstrates an average crystallite size of roughly 28 nm. Consequently, the wear rate measured for the former coating is lower than that of the latter. When the surface of a tribological component is coated with a material that possesses much greater hardness compared to the component itself, the wear experienced by the component is substantially lowered. This reduction in wear may be attributed to the inherent property of hard materials to exhibit lower rates of wear when subjected to same frictional circumstances, in comparison to softer materials [22].

Figure 6c illustrates the impact of load on the wear rate of uncoated brass pins and two types of TiVN-coated brass pin samples, namely, TiVN-325 and TiVN-450. The data presented indicate a noticeable disparity in wear rates between uncoated brass pins and TiVN-coated brass pins when subjected to equal wear circumstances. The wear rate of vanadium samples, namely, TiVN-450 and TiVN-325 coatings, as well as brass pins, falls within the range of 1.76 × 10^−3^ to 5.87 × 10^−3^ mm^3^/Nm, with variations based on the applied stress. When the applied force is raised from 10 to 40 N, the rate of wear for the uncoated brass pin exhibits an increase from 3.51 × 10^−3^ to 5.87 × 10^−3^ mm^3^/Nm. Similarly, for the vanadium sample with TiVN-450 coating, the wear rate increases from 2.02 × 10^−3^ to 4.97 × 10^−3^ mm^3^/Nm, while for the TiVN-325 coating, the wear rate increases from 1.76 × 10^−3^ to 4.88 × 10^−3^ mm^3^/Nm.

Figure 6d illustrates the impact of sliding distance on the rate of wear for both uncoated and TiVN-coated brass pins from samples TiVN-450 and TiVN-325. The wear rate of uncoated brass pins exhibits a rise in correlation with the sliding distance, ranging from 3.51 × 10^−3^ to 1.19× 10^−2^ mm^3^/Nm. Similarly, for the vanadium sample with TiVN-450 coating, the wear rate increases from 2.02 × 10^−3^ to 9.1 × 10^−3^ mm^3^/Nm, while for the TiVN-325 coating, it increases from 1.76 × 10^−3^ to 8.86 × 10^−3^ mm^3^/Nm. The tribological characteristics of TiVN were investigated by Ouyang and Sasaki. The researchers made an observation that the wear rate of the coating exhibits an increase from 4.72 × 10^−8^ to 8.42 × 10^−8^ mm^3^/Nm as the load is varied within the range of 20 to 100 N [21]. The wear rate of a brass pin coated with TiVN is lower in comparison to an uncoated brass pin, and these findings align with those described in the existing literature.

**Figure 7:**
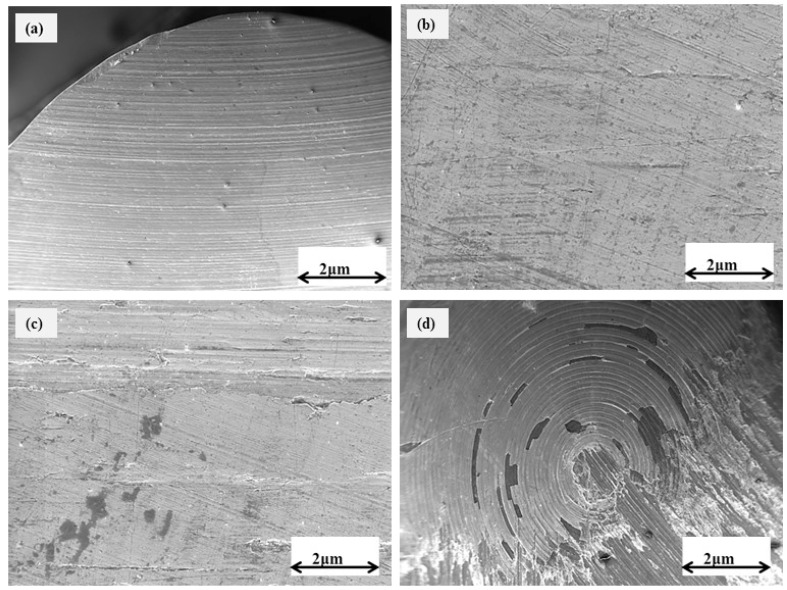
SEM images of uncoated brass pins at a load of (**a**) 10, (**b**) 20, (**c**) 30, and (**d**) 40 N.

**Figure 8:**
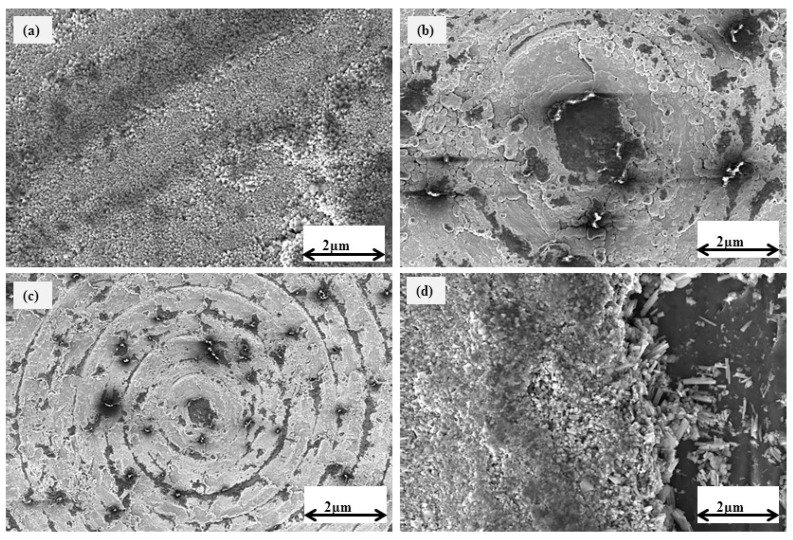
SEM images of TiVN-coated brass pins at a load of (**a**) 10, (**b**) 20, (**c**) 30, and (**d**) 40 N.

Figure 7 and Figure 8 depict the scanning electron microscope (SEM) pictures of the worn surfaces of both uncoated and TiVN-coated brass pins. The wear rate of the uncoated brass pin is seen to be greater when the applied load is raised in increments of 10 N at values of 10, 20, 30, and 40 N, as depicted in Figure 7a–d accordingly. SEM images of TiVN-coated brass pins under different load conditions are presented in Figure 8a–d. The loads applied during the imaging process were 10, 20, 30, and 40 N, respectively. The worn surface of TiVN-coated brass pins exhibits dark regions at a 10 and 20 N load, as seen in Figure 8a,b, respectively. When a load of 30 and 40 N is exerted on brass pins coated with TiVN, the resulting surface damage exhibits rough scratching. Additionally, dark areas are present, along with many thin needle-like rolls. Figure 8c,d depict these observations, including the presence of white particles on the rolls. The brass pins possess the capability to maintain a TiVN coating, which effectively mitigates friction and wear in comparison to brass pins lacking such a coating. This advantage is observed even under demanding tribological testing circumstances, namely, at a load of 40 N at a rotational speed of 500 rpm.

## 4. Conclusions

The deposition of titanium vanadium nitride films was achieved by the utilization of reactive DC magnetron sputtering on brass substrates. This was accomplished by altering the Ar:N_2_ gas ratio and adjusting the power levels of vanadium. The X-ray diffraction (XRD) patterns provided evidence for the development of (220), (111), and (222) crystallographic orientations in the TiVN coatings when the nitrogen:argon (N_2_:Ar) gas ratio increased. However, when the vanadium power levels were altered, only the (111) and (222) peaks were seen. The study revealed a clear correlation between the wear rate and coefficient of friction, and the fluctuations in gas ratio and power levels. The brass pins coated with TiVN demonstrate a range of friction coefficients, namely, between 0.19 and 0.31, in response to alterations in the gas ratio. Conversely, when subjected to changes in power levels, the measured friction coefficient at room temperature varies between 0.18 and 0.30, depending on the load and sliding distance. The wear rate of uncoated brass has a greater magnitude when compared to the TiVN coatings when subjected to equal wear circumstances. The wear rates of the TiVN coatings and brass exhibit a range of 1.76 × 10^−3^ to 9.14 × 10^−4^ mm^3^/Nm, which is contingent upon factors such as load, sliding distance, gas ratio, and vanadium power.

## Data Availability

Not applicable.
